# School food programs and food insecurity at the REACH school network: an observational study

**DOI:** 10.1186/s12889-025-23163-8

**Published:** 2025-06-03

**Authors:** Kavin Qiu, Jessica A. Omand, Youssef Elshaarawi, Saisujani Rasiah-Shaidev, Charles Keown-Stoneman, Justine Cohen-Silver, Jonathon.L Maguire, Sloane Jaye Freeman

**Affiliations:** 1 Women and Children’s Health Program, St. Michael’sHospital, Unity Health Toronto, 30 Bond Street 15 CC S, Toronto, ON M5B1W8 Canada; 2 School of Nutrition - Faculty of Community Services, Toronto Metropolitan University, Daphne Cockwell Health Sciences Complex, 288 Church St., Room DCC-601, Toronto, ON Canada; 3 Applied Health Research Centre, St. Michael’s Hospital, 30 Bond Street, Toronto, ON M5B1W8 Canada; 4 Women and Children’s Health Program St. Joseph’s Health Centre, Unity Health Toronto, 30 The Queensway, Toronto, Ontairo M6R 1B5 Canada; 5 Women and Children’s Health Program, St. Michael’s Hospital, 2nd Floor Pediatric Clinic Unity Health Toronto, Toronto, ON M4C 2T2 Canada; 6 Li Ka Shing Knowledge Institute, St. Michael’s Hospital, Unity Health Toronto, Toronto, Canada; 7 Dalla Lana School of Public Health, University of Toronto, Toronto, Canada

**Keywords:** Food insecurity, School food programs, School meals, Nutrition, Equity, Policy

## Abstract

**Background:**

Food insecurity is common in Canada and impacts children more than any other age group. This study aimed to evaluate the association between participation in school food programs and food insecurity among students attending Canada’s largest urban school-based health centre program, the REACH School Network.

**Methods:**

This is a cross-sectional observational study from April 2022 to June 2024 at the REACH School Network. We administered the Growth and Nutrition Questionnaire to parents of children aged 3–17 years. Questions were related to dietary intake and participation in school food programs. Our primary outcome was food insecurity, using the Hunger Vital Sign. Logistic regression estimated the association between regular school food program participation and food insecurity, adjusting for covariates.

**Results:**

Of 477 eligible participants, 316 consented (66.2% response rate), and 223 were included in the analysis. The mean age was 9.21 years (SD = 3.08); 69.1% identified as male. Overall, 134 (60.1%) regularly participated in a school food program. Food insecurity was reported by 97 (43.5%) participants, with similar prevalence among participants (44.8%) and non-participants (41.6%). Logistic regression, both unadjusted (OR = 1.14; 95% CI 0.66–1.97; *P* = 0.637) and adjusted (OR = 0.82; 95% CI 0.42–1.63; *P* = 0.579) found no significant association between school food program participation and food insecurity.

**Conclusion:**

Our study highlights the complex relationship between food insecurity and school food program participation among an at-risk, urban children. Future research is needed, with larger sample sizes and longitudinal designs to better understand these complex relationships.

**Supplementary Information:**

The online version contains supplementary material available at 10.1186/s12889-025-23163-8.

## Background

Food insecurity is common in Canada and the United States (US), with reported rates of 22.9% and 13.5% in 2023, respectively [1, 2]. Food insecurity impacts Canadian children more than any other age group, with rates as high as 28.4% [2–5]. Food insecurity has been linked to poor nutrition, growth and development, as well as mental health conditions, behaviour problems, and obesity among children [6]. 

Since children typically spend a third of their waking-hours in school, school food programs (SFP)s play a key role in providing consistent and nutritious food to children, promoting better health and academic outcomes, especially for children from low-income households who may face greater barriers to accessing adequate nutrition [7–9]. In Canada, one in five children participate in SFPs [10]. In the US, close to 60% of students participate in the national school lunch program [11]. Overall, over 28.6 million students rely on SFPs in the US, especially those with social inequities [12]. Canada’s SFPs are largely dependent on community partnerships and local government support, with significant disparities in program availability and reach across different jurisdictions. A recent Canada-wide survey of school food programs revealed a highly variable and fragmented landscape, with SFPs differing considerably in their structure, goals, and sources of funding [13]. While many organizations reported reducing student food insecurity as a key objective, the majority of programs relied primarily on donations, with provincial funding accounting for only 30% of total financial support [13]. Nutrition and cost were cited as primary factors influencing food selection [13]. These findings underscore the limitations of Canada’s current patchwork approach and the potential benefits of a cohesive national strategy. In June 2024, the Government of Canada released its National School Food Policy and pledged $1 billion toward the development and implementation of a nationally harmonized SFP that could help close these gaps [24]. 

Countries with national SFPs, such as the US, are primarily government funded [14]. In addition to providing meals at no cost to students, SFPs allow families the autonomy to direct the savings to other expenses, which may further reduce food insecurity [15]. SFPs are associated with a reduction in obesity and improved academic outcomes, as well as reduced healthcare spending [16–18]. Participation in both the US Department of Agriculture’s National School Lunch Program and School Breakfast Program has been shown to provide close to 60% of children’s daily food intake, acting as a nutrition safety net for low-income children [19]. In Sweden, school lunches have been shown to provide around half of children’s daily vegetable intake and two-thirds of their daily fish intake [20]. Japan’s national lunch program has improved student’s total dietary quality, especially for vitamins and minerals [21]. In contrast to Canada’s historically decentralized model, these countries’ programs are primarily government-funded and operate on a universal basis, providing meals at no cost and functioning as a crucial nutritional safety net for children.

While research has shown an association between SFPs and improved health and academic outcomes, the association between SFPs and food insecurity remains unclear [17, 18]. The objective of this study was to evaluate the association between participation in SFPs and food insecurity among students attending Canada’s largest urban school-based health centre program. We hypothesised that students who regularly participated in SFPs would experience lower food insecurity compared to students without access.

## Methods

### Study setting

We conducted a cross-sectional study between April 2022 and June 2024 with a convenience sample of children aged 3 to 17 years who attended the REACH School Network. The REACH School Network is a school-based health centre program with two school-based health centres serving at-risk students from 180 elementary schools in low-income neighbourhoods within the Toronto District School Board. Children were referred to the REACH School Network by parents or teachers for developmental and mental health concerns. All children attending the REACH School Network were eligible for the study. However, children who were home-schooled in the year preceding the time of contact were excluded from the study. Parents were invited to participate in a de-identified, self-administered questionnaire either in-person or over the phone by a research assistant.

### Measures

We administered a parent-reported Growth and Nutrition Questionnaire adapted from the TARGet Kids! Nutrition and Health Questionnaire, which is based on the Canadian Community Health Survey [22–24]. The questionnaire was comprised of questions related to dietary intake and the food environment including; household meals, and food insecurity as measured by the Hunger Vital Sign [25]. Parents were asked about how often their child participated in their home school’s SFPs. Participation in SFPs was determined by the frequency of the child’s participation with options being “Always”, “Usually”, “About half the time”, “Rarely”, or “Never”. This question was posed for three SFPs: a morning meal or breakfast program, a snack program, and a lunch program. Responses indicating “Always”, “Usually” and “About half the time” for at least one of the above SFPs were categorized as regular participation. The Growth and Nutrition Questionnaire was translated to French, Chinese, Arabic, and Urdu as these were the most frequent languages spoken by families other than English (Supplementary Material). Sociodemographic data including family income, parental employment status, ethnicity, and newcomer status were also collected. Due to low numbers in each ethnicity category as it was collected, ethnicity was collapsed into the following categorizes for the analyses, “African and Caribbean”, “East and South East Asian”, “European”, “Mixed”, “North American”, “Other”, “South Asian” (Table 1). The full list of ethnicity categories are included as Supplementary Material, Table 1.

**Table 1:** Overall and by regular participation in school food programs^a^

	Level	Overall *N* = 223	No *N* = 89	Yes *N* = 134	Standardized mean difference (SMD)
Age (years)		9.21 (3.08)	9.33 (3.32)	9.13 (2.92)	0.063
Age categories (years)	0–7	90 (40.4)	41 (46.1)	49 (36.6)	0.486
	8–11	87 (39.0)	23 (25.8)	64 (47.8)	
	12+	46 (20.6)	25 (28.1)	21 (15.7)	
Gender	Female	68 (30.5)	29 (32.6)	39 (29.1)	0.142
	Male	154 (69.1)	60 (67.4)	94 (70.1)	
	Other Gender Identity	1 (0.4)	0 (0.0)	1 (0.7)	
Food insecurity	No	126 (56.5)	52 (58.4)	74 (55.2)	0.065
	Yes	97 (43.5)	37 (41.6)	60 (44.8)	
Ethnicity collapsed	African and Caribbean	32 (15.3)	10 (12.0)	22 (17.5)	0.373
	East and South East Asian	17 (8.1)	10 (12.0)	7 (5.6)	
	European	44 (21.1)	17 (20.5)	27 (21.4)	
	Mixed	51 (24.4)	17 (20.5)	34 (27.0)	
	North American	14 (6.7)	6 (7.2)	8 (6.3)	
	Other	23 (11.0)	8 (9.6)	15 (11.9)	
	South Asian	28 (13.4)	15 (18.1)	13 (10.3)	
Family income	< $30,000	55 (24.7)	21 (23.6)	34 (25.4)	0.215
	$30,000 to $49,999	24 (10.8)	12 (13.5)	12 (9.0)	
	$50,000 to $74,999	27 (12.1)	11 (12.4)	16 (11.9)	
	$75,000 to $99,999	16 (7.2)	8 (9.0)	8 (6.0)	
	>$100,000	31 (13.9)	11 (12.4)	20 (14.9)	
	does not know	10 (4.5)	3 (3.4)	7 (5.2)	
	No information	60 (26.9)	23 (25.8)	37 (27.6)	
Parent 1 education	College/university	146 (65.5)	58 (65.2)	88 (65.7)	0.197
	Elementary school	6 (2.7)	2 (2.2)	4 (3.0)	
	High school	55 (24.7)	21 (23.6)	34 (25.4)	
	No formal schooling	1 (0.4)	0 (0.0)	1 (0.7)	
	No information	15 (6.7)	8 (9.0)	7 (5.2)	
Parent 2 education	College/university	100 (44.8)	42 (47.2)	58 (43.3)	0.303
	Elementary school	7 (3.1)	1 (1.1)	6 (4.5)	
	High school	36 (16.1)	12 (13.5)	24 (17.9)	
	No formal schooling	4 (1.8)	3 (3.4)	1 (0.7)	
	No information	76 (34.1)	31 (34.8)	45 (33.6)	

^a^For numeric variables Mean (SD) are reported, for categorical variables, N (%) are reported

Our primary outcome was food insecurity, as collected through the Hunger Vital Sign, a validated 2-question food insecurity screening tool derived from the US Department of Agriculture’s Household Food Security Survey [25]. The Hunger Vital Sign has been used in the pediatric population to assess food insecurity [26]. The Hunger Vital Sign identifies households as being at risk for food insecurity if they respond with “often true” or “sometimes true” (as opposed to “never true”) to either or both of the following two statements: “Within the past 12 months we worried whether our food would run out before we got money to buy more,” and “Within the past 12 months the food we bought just didn’t last and we didn’t have money to get more?” This study adhered to the STROBE statement [27]. 

### Analysis

A sample size calculation was not conducted as this study was based on a convenience sample. Logistic regression was used to estimate the unadjusted association between regular participation in a SFP and being at risk for food insecurity. An adjusted logistic regression model was also fit to estimate the adjusted association between regular participation in a SFP and being at risk for food insecurity, accounting for child age, gender, ethnicity, and family income. For the adjusted model, multivariate multiple imputations were used for missing covariate data [28, 29]. Specifically, 50 multivariate imputed data sets were generated, with missing values in ethnicity and family income imputed. The imputation model included information on the other adjusting variables in the final model (i.e., age and gender) as well as parental education from both parents (if available). Due to separation or quasi-separation of some of the adjusting variables and the outcome (for example, no child in the ‘>$100,000’ family income category reported experiencing food insecurity), a Firth penalized likelihood was used for the logistic regression [30]. All analyses were performed using R version 4.4.2 for Windows 64bit [31]. 

## Results

**Fig. 1:**
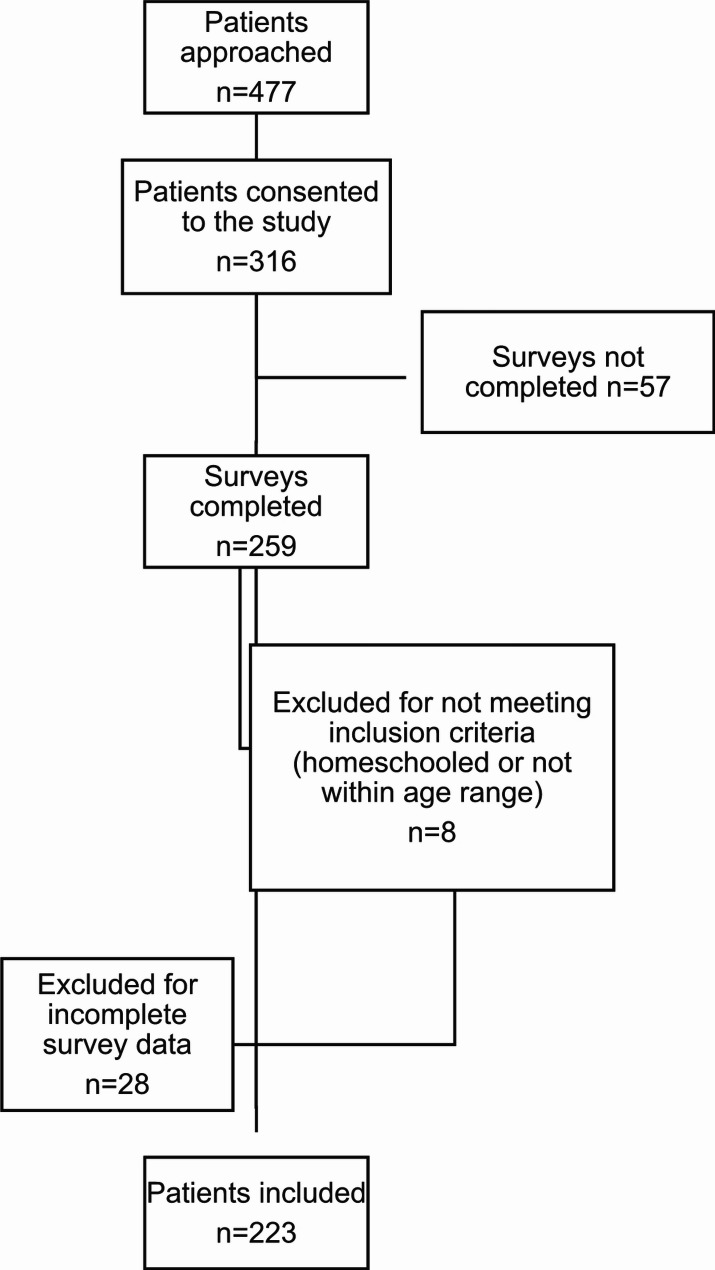
Study participant flow diagram

A total of 477 participants were approached for the study and 316 consented to participate (66% response rate). A total of 259 participants completed the survey, 36 participants were excluded for incomplete data or not meeting the inclusion criteria, and 223 children were included in the analysis (Fig. 1).

The mean age of participants was 9.21 years (SD = 3.08). Most participants identified as male (69.1%), followed by female (30.5%) and other (0.4%). Sociodemographic characteristics for those who participated in SFPs and those did not participate are reported in Table 1. Of the 223 participants, 134 (60.1%) regularly participated in a SFP, while 89 (39.9%) did not. In the imputed process, there were no missing values to impute in the outcome (food insecurity), the exposure (regular participation in SFPs), age, or gender. There were 14 subjects missing ethnicity (6.3%), 70 missing family income (31.4%), 15 missing education for the first parent (6.7%), and 76 missing education for the second parent (34.1%) (Table 1).

Food Insecurity.

Overall, 97 participants (43.5%) reported food insecurity. The prevalence of food insecurity was higher among those who regularly participated in SFPs (44.8%) compared to those who did not (41.6%). In the unadjusted analysis there was insufficient evidence that regular participation in SFPs was associated with food insecurity (OR = 1.14; 95% CI 0.66–1.97; *P* = 0.637; Table 2). In the primary analysis, adjusting for age, gender, family income and ethnicity, there was insufficient evidence that regular participation in SFPs was associated with food insecurity (OR = 0.82; 95% CI 0.42–1.63; *P* = 0.579; Table 3).

**Table 2:** Unadjusted logistic regression for odds of food insecurity

	Odds-ratio	Lower 95% CI	Upper 95% CI	*p*-value
exp(Intercept)	0.712	0.464	1.081	0.114
Regular participation in school food program	1.140	0.664	1.965	0.637

**Table 3:** Adjusted logistic regression for odds of food insecurity

	Odds-ratio	Lower 95% CI	Upper 95% CI	*p*-value
exp(Intercept)	18.758	3.509	100.270	0.001
Regular participation in school food program	0.824	0.415	1.634	0.579
Age (Years)	0.961	0.858	1.078	0.500
Gender: Male (Ref)	1.000	-	-	-
Gender: Female	1.203	0.583	2.482	0.618
Gender: Other gender identity	0.265	0.008	8.343	0.450
Ethnicity: Mixed (Ref)	1.000	-	-	-
Ethnicity: African and Caribbean	0.365	0.090	1.484	0.159
Ethnicity: East and South East Asian	0.103	0.021	0.507	0.005
Ethnicity: European	0.176	0.044	0.696	0.013
Ethnicity: North American	0.125	0.025	0.622	0.011
Ethnicity: Other	0.112	0.026	0.486	0.003
Ethnicity: South Asian	0.093	0.023	0.385	0.001
Family income: >$30,000 (Ref)	1.000	-	-	-
Family income: $30,000 to $49,999	0.390	0.144	1.058	0.065
Family income: $50,000 to $74,999	0.360	0.140	0.922	0.033
Family income: $75,000 to $99,999	0.028	0.005	0.163	< 0.001
Family income: $100,000+	0.006	< 0.001	0.079	< 0.001

## Discussion

This cross-sectional observational study of 223 children at the REACH School Network did not find evidence of an association between regular participation in SFPs and food insecurity. While the prevalence of food insecurity was high overall (43.5%), and slightly higher among those who regularly participated in SFPs (44.8%) compared to those who did not (41.6%), these differences were not statistically significant. Given the sampling variability, relatively small sample size, and lack of adjustment for potential confounders, these findings should be interpreted with caution.

Our results are similar to Yang et al. who found a higher prevalence of food insecurity among children receiving free school meals through the National School Lunch Program in the US compared to those who did not during the COVID-19 pandemic [32]. Similarly, another study which investigated the National School Lunch Program prior to the COVID-19 pandemic, did not find effects of SFP participation on food insecurity [33]. Our findings contrast a study which demonstrated that access to the national School Breakfast Program in the US reduced marginal food insecurity among children from low-income households [34]. The lack of an observable impact on food insecurity may be attributed to both the cross-sectional nature of our study and the sample size relative to the potential effect size, which may have limited our ability to detect a significant association. A longitudinal study examining the DIATROFI program in Greece, suggested that the effectiveness of SFPs in reducing food insecurity may increase with sustained participation over multiple years [35]. 

The prevalence of food insecurity observed in our study population is higher than reported rates in Toronto, Ontario, and Canada [3, 36]. A report from Public Health Ontario in 2019 highlighted that 18.5% of children had food insecurity in Toronto, higher than the provincial average of 20.6% of food insecure households indicated by the 2020 Canadian Income Survey, and lower than the national proportion of 28.4% reported by Statistics Canada in 2022 [3, 36]. These rates are even more pronounced among low-income, inner-city populations, as reflected in our findings. Our findings could be attributable to the rising cost of living, inflation, and other systemic barriers that disproportionately impact at-risk families [37]. 

Regular participants in SFPs in our study were slightly younger compared to non-participants, and the majority identified as African, European, or mixed ethnicities. Similarly, a study by Zuercher et al. found that among those accessing school lunch programs in California, White, Black, and Hispanic students consumed school lunches more frequently than students in the other race/multiracial group [38]. 

While SFPs are valuable, they may act more as a buffer than a remedy, especially in the short term. These programs can help alleviate some of the immediate challenges faced by low-income families by reducing grocery costs, ensuring children have access to nutritious meals during school hours, and supporting academic performance [9, 39, 40]. However, additional strategies are required to mitigate the broader structural determinants of food insecurity, such as poverty, unemployment, and racial discrimination [41]. Additionally, the fragmented and locally administered nature of SFPs in Canada—as opposed to the more comprehensive, nationally funded models in countries like the US, Sweden, and Japan—may limit their effectiveness in addressing systemic disparities. Broader interventions—such as income supports, affordable housing initiatives, and policies addressing the affordability and accessibility of healthy food—are necessary to complement SFPs and achieve meaningful reductions in food insecurity [42–45]. 

### Implications for policy and practice

Our findings have several implications for policy and practice. First, the fragmented nature of SFP funding and administration in Canada calls for greater federal investment and standardization to ensure equitable access and consistent quality across provinces. A national school food program, modelled after successful initiatives in other countries, could provide a more robust safety net for low-income families. Efforts have begun to develop in this area, with a commitment to a national SFP by the Canadian government. Second, efforts to reduce stigma associated with SFP participation should be prioritized, such as universal meal programs that normalize participation regardless of socioeconomic status. Additionally, policies that provide direct financial support to low-income families—such as expanded child benefits or living wage initiatives—could complement the role of SFPs in reducing food insecurity.

### Strengths and limitations

One of the key strengths of our study is the use of a validated parent-reported food security screening tool, which allowed us to assess food insecurity status. Additionally, our study has benefited from the inclusion of sociodemographic characteristics which accounted for potential confounders such as household income and ethnicity. Furthermore, the study setting of the REACH School Network has facilitated access to a population that is often underrepresented in research, ensuring that our findings reflect the experiences of those most affected by food insecurity.

Our study also had several limitations that should be considered when interpreting the findings. First, this study used self-reported data for SFP participation and food insecurity, which may be subject to recall or social desirability bias [46]. Additionally, while the Hunger Vital Sign is a validated measure of food insecurity among children, it is limited in its scope and lacks important aspects of food insecurity such as, access to balanced meals, access to a variety of foods, skipping meals, portion size, and experiences with hunger. Furthermore, the cross-sectional and observational nature of this study limited our ability to establish causality or determine the directionality of the observed associations. We were unable to assess the effects of sustained participation in SFPs over time, which is important for understanding the long-term impact of these programs on food insecurity. Our sample size was also relatively small, which may have reduced the statistical power to detect clinically meaningful associations. Furthermore, our findings were derived from a single urban cohort in Toronto and may not be generalizable to other regions with differing socioeconomic contexts or SFP structures. For instance, rural communities face unique barriers to food access, which may influence the relationship between SFPs and food insecurity in ways not captured by our data [47]. Additionally, the observed lack of association may be influenced by unmeasured confounders such as employment status, household size, or housing stability. Lastly, insights into the lived experiences of families navigating food insecurity or logistical challenges faced by schools in implementing SFPs could provide valuable context and inform targeted interventions through the collection of qualitative data in a future study.

## Conclusions

Our study highlights the complex relationship between food insecurity and SFP participation among an at-risk, urban children who were participating in the REACH School Network. SFPs may require broader supports to reduce household food insecurity. Future research is needed, with larger sample sizes and longitudinal designs to better understand these complex relationships.

## Electronic supplementary material

Below is the link to the electronic supplementary material.

Supplementary Material 1

Supplementary Material 2

## Data Availability

The datasets used and/or analyzed during the current study are available from the corresponding author on reasonable request.

## Abbreviations

SFP: School food program
OR: Odds ratio
CI: Confidence interval
US: United States
