# Supplementary material for: School food programs and food insecurity at the REACH school network: an observational study
Source: BMC Public Health. 2025 Jun 3;25:2060. doi: 10.1186/s12889-025-23163-8 (PMC12131624; doi:10.1186/s12889-025-23163-8)
Supplement: Supplementary file 1 — Supplementary Material 1 [file 12889_2025_23163_MOESM1_ESM.docx]

School Based Health Centre
 **Growth and Nutrition Questionnaire** Spruce/Nelson Mandela/Parkdale
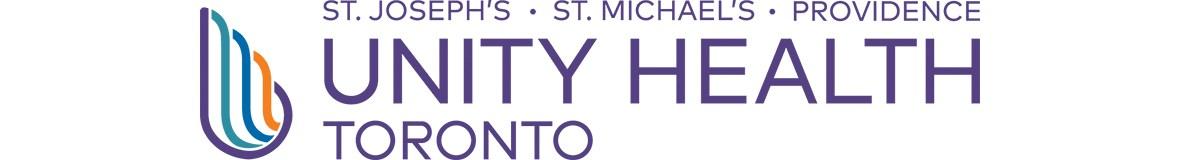


**Participant ID#: ________**

Thank you for taking the time to complete the following questionnaire regarding your child’s growth and nutrition. If you have any questions, feel free to ask any of the study staff. **Your privacy is highly respected by our study team, all information will remain confidential.**

**Dietary intake**

1a) My child usually eats fruit

|  |  | More than 3 times a day |
| --- | --- | --- |
|  |  | 3 times a day |
|  |  | 2 times a day |
|  |  | Once a day |
|  |  | Not at all |

1b) My child usually eats vegetables

|  | More than 2 times a day |
| --- | --- |
|  | 2 times a day |
|  | Once a day |
|  | Not at all |

| 1c) Circle how many **cups** your child drinks in a typical day: |
| --- |

| **Liquid** | **Portion size** | | | | | | |
| --- | --- | --- | --- | --- | --- | --- | --- |
|  | **(1 cup = 8 ounces = 250 ml)** | | | | | | |
| Cow’s milk - white | 0 | ½ | 1 | 2 | 3 | 4 | 5+ |
| Cow’s milk - chocolate | 0 | ½ | 1 | 2 | 3 | 4 | 5+ |
| Milk alternatives | 0 | ½ | 1 | 2 | 3 | 4 | 5+ |
| 100% Juice (e.g., apple, orange, grape, prune) | 0 | ½ | 1 | 2 | 3 | 4 | 5+ |
| Sweetened drinks (e.g., Kool aid, iced tea, Gatorade, fruit punch) | 0 | ½ | 1 | 2 | 3 | 4 | 5+ |
| Soda or pop | 0 | ½ | 1 | 2 | 3 | 4 | 5+ |

**Food Environment**

| 2a) In a typical week, how many times does your family eat the breakfast meal together? |  | days/week |
| --- | --- | --- |

| 2b) In a typical week, how many times does your family eat the evening meal together? |  | days/week |
| --- | --- | --- |

| 3a) How many meals does your child have each day? |  | | # of meals | |  |
| --- | --- | --- | --- | --- | --- |
|  |  |  | |  |  |
| 3b) How many snacks does your child have each day? |  | | # of snacks | | |

| 3c) For the main meal of the day does your child eat a different meal from that eaten by other members of the family: | | |  |
| --- | --- | --- | --- |
|  |  | Always | |
|  |  | Almost always | |
|  |  | Sometimes | |
|  |  | Almost never | |
|  |  | Never | |

| 3d) | For the main meal of the day does your child eat store-bought packaged food (e.g., frozen, canned): |
| --- | --- |
|  | Always |
|  | Almost always |
|  | Sometimes |
|  | Almost never |
|  | Never |

| 3e) | Do you feel that you have had difficulties feeding your child in the past year? |
| --- | --- |
|  | Yes, great difficulty |
|  | Yes, some difficulty |
|  | No difficulties |

| 3f) | In the past year, has your child at any time: | | | | | | | | |
| --- | --- | --- | --- | --- | --- | --- | --- | --- | --- |
|  | |  | Often |  | Sometimes |  | Rarely |  | Never |
| Not eaten sufficient amount of food | |  |  |  |  |  |  |  |  |
| Refused to eat the right food | |  |  |  |  |  |  |  |  |
| Been picky with food | |  |  |  |  |  |  |  |  |
| Overeaten | |  |  |  |  |  |  |  |  |
| Been difficult to get into an eating routine | |  |  |  |  |  |  |  |  |

| 4a) | How does your child react to new foods? Do they: |
| --- | --- |
|  | Willingly tries new foods |
|  | Have to be convinced to try new foods but generally accepts them |
|  | Generally resists new foods |

| 4b) | Which best describes your child’s acceptance of different food textures? |
| --- | --- |
|  | Willingly eats a number of different food textures |
|  | Resists eating certain food textures |
|  | Refuses to eat certain food textures |

| 5a) | “Within the past 12 months we worried whether our food would run out before we got money to buy more”. Was that often true, sometimes true, or never true for  (you/your household) **in the last 12 months**. |
| --- | --- |
|  | Often true |
|  | Sometime true |
|  | Never true |

| 5b) | “Within the past 12 months the food we bought just didn’t last and we didn’t have money to get more”. Was that often true, sometimes true, or never true for  (you/your household) **in the last 12 months.** |
| --- | --- |
|  | Often true |
|  | Sometime true |
|  | Never true |

**Questions pertaining to school food programs:**

6a) Does your child’s school have a funded school food program?

| **Does your school have a funded Program listed below** | **Have you participated in this program?** | **Please specify whether your child participates in this program.** | **Does your child pay for this program?** |
| --- | --- | --- | --- |
| Funded school breakfast program (breakfast or morning meal) |  Yes   No   Don’t Know |  Always   Usually   About half the time   Rarely   Never |  Yes   No |
| Funded school snack program |  Yes   No   Don’t Know |  Always   Usually   About half the time   Rarely   Never |  Yes   No |
| Funded school lunch program |  Yes   No   Don’t Know |  Always   Usually   About half the time   Rarely   Never |  Yes   No |
|  No funded school food program available |  |  |  |
|  I do not know |  |  |  |

6b) What would you like to see offered more in your child’s funded school food program? Select all that apply.

- Fresh fruit
- Fresh vegetables
- Cow’s milk
- Milk alternatives (soy, almond, rice, goat milk etc.)
- Meat and alternatives
- Vegetarian/plant-based options
- Dairy free
- Gluten free
- Cultural foods
- Lower fat options
- Lower carbohydrate options
- Other, **please specify**: _________________________________
- Not applicable

7) Where does your child usually get lunch on school days? Select all that apply.

| Your child brings lunch from home:  Always   Usually   About half the time   Rarely   Never | Your child buys lunch off school campus:   Always   Usually   About half the time   Rarely   Never |
| --- | --- |
| Your child goes home for lunch:   Always   Usually   About half the time   Rarely   Never | Your child purchases snacks from a vending machine for lunch:   Always   Usually   About half the time   Rarely   Never |
| Your child gets lunch from the school cafeteria:   Always   Usually   About half the time   Rarely   Never | Your child does not like to eat lunch at school (they skip lunch):   Always   Usually   About half the time   Rarely   Never |
| Your child gets lunch delivered from an offsite vendor (not including the funded school lunch program):   Always   Usually   About half the time   Rarely   Never |  I do not know |

8a) On a typical day, my child helps to pack their own lunch.

- Always
- Usually
- About half the time
- Rarely
- Never

8b) On a typical day, what percentage of your child’s packed school lunch comes home with them (i.e., they don’t eat it at school): _________%

**Education outcomes**

| 9) What grade is your child in? | |
| --- | --- |
|  | Junior Kindergarten |
|  | Senior Kindergarten |
|  | Grade 1 |
|  | Grade 2 |
|  | Grade 3 |
|  | Grade 4 |
|  | Grade 5 |
|  | Grade 6 |
|  | Grade 7 |
|  | Grade 8 |
|  | Grade 9 |
|  | Grade 10 |
|  | Grade 11 |
|  | Grade 12 |

| 10) How does your child attend school? | |
| --- | --- |
|  | In-person |
|  | Virtual |
|  | Mixed in-person and virtual |
| If mixed, what proportion of school time was in person? ______% | |

| 11) | Since the start of the school year, how many days has your child been absent from their school (either in-person or virtual)? _______________ days |
| --- | --- |

| 12) Based on your knowledge of your child’s most recent report card, how well is your child doing in the following subjects? | | | | | | |
| --- | --- | --- | --- | --- | --- | --- |
|  | **A**  **(Level 4)** | **B**  **(Level 3)** | **C**  **(Level 2)** | **D**  **(Level 1)** | **F or R (Remedial)** | **N/A** |
| 1. Language (including Reading, Writing and Oral communication) | **☐** | **☐** | **☐** | **☐** | **☐** | **☐** |
| 1. Mathematics | **☐** | **☐** | **☐** | **☐** | **☐** | **☐** |
| 1. Science and Technology | **☐** | **☐** | **☐** | **☐** | **☐** | **☐** |
| 1. Social Studies (including History or Geography) | **☐** | **☐** | **☐** | **☐** | **☐** | **☐** |
| 1. Health and Physical Education | **☐** | **☐** | **☐** | **☐** | **☐** | **☐** |
| 1. The Arts | **☐** | **☐** | **☐** | **☐** | **☐** | **☐** |
| 1. French | **☐** | **☐** | **☐** | **☐** | **☐** | **☐** |

| 13) Does your child receive an Individual Education Plan (IEP)? | |
| --- | --- |
|  | No |
|  | Yes – **Please describe**: __________________________________________ |

14) During this school year, how would you rate the following?

|  | **Strongly agree** | **Agree** | **Neither agree nor disagree** | **Disagree** | **Strongly disagree** | **Don’t know** |
| --- | --- | --- | --- | --- | --- | --- |
| My child is doing well in school | **☐** | **☐** | **☐** | **☐** | **☐** | **☐** |
| My child is well prepared for next year | **☐** | **☐** | **☐** | **☐** | **☐** | **☐** |
| My child is good at mathematics | **☐** | **☐** | **☐** | **☐** | **☐** | **☐** |
| My child likes mathematics | **☐** | **☐** | **☐** | **☐** | **☐** | **☐** |
| My child is a good reader | **☐** | **☐** | **☐** | **☐** | **☐** | **☐** |
| My child likes to read | **☐** | **☐** | **☐** | **☐** | **☐** | **☐** |
| My child is a good writer | **☐** | **☐** | **☐** | **☐** | **☐** | **☐** |
| My child likes to write | **☐** | **☐** | **☐** | **☐** | **☐** | **☐** |
| My child is getting good grade/marks | **☐** | **☐** | **☐** | **☐** | **☐** | **☐** |

15) During the **LAST MONTH**, how would you rate the following?

|  | **Strongly agree** | **Agree** | **Neither agree nor disagree** | **Disagree** | **Strongly disagree** | **Don’t know** | **Does not apply** |
| --- | --- | --- | --- | --- | --- | --- | --- |
| My child enjoys school | **☐** | **☐** | **☐** | **☐** | **☐** | **☐** | **☐** |
| My child is bored at school | **☐** | **☐** | **☐** | **☐** | **☐** | **☐** | **☐** |
| My child is interested in the tasks and assignments they are asked to complete | **☐** | **☐** | **☐** | **☐** | **☐** | **☐** | **☐** |
| My child gets involved/participates in class discussions | **☐** | **☐** | **☐** | **☐** | **☐** | **☐** | **☐** |

**Thank you for taking the time to complete this questionnaire! We appreciate it.**
