# Supplementary material for: School food programs and food insecurity at the REACH school network: an observational study
Source: BMC Public Health. 2025 Jun 3;25:2060. doi: 10.1186/s12889-025-23163-8 (PMC12131624; doi:10.1186/s12889-025-23163-8)
Supplement: Supplementary file 2 — Supplementary Material 2 [file 12889_2025_23163_MOESM2_ESM.docx]

**Supplementary Material**

**Table 1.** Full list of ethnicities

|  | **Level** | **Overall N=223** | **No**  **N=89** | **Yes**  **N=134** | **Standardized mean difference (SMD)** |
| --- | --- | --- | --- | --- | --- |
| Ethnicity | 1=North American (e.g., Canadian, American, etc. ) | 14 ( 6.3) | 6 ( 6.7) | 8 ( 6.0) | 0.461 |
|  | 2=South Asian (e.g., East Indian, Pakistani, Sri Lankan, etc.) | 28 (12.6) | 15 (16.9) | 13 ( 9.7) |  |
|  | 3=East Asian (e.g., Chinese, Mongolian, Korean, Japanese, etc.) | 10 ( 4.5) | 5 ( 5.6) | 5 ( 3.7) |  |
|  | 4=African (e.g., Ghanaian, Somalian, South African, etc.) | 12 ( 5.4) | 2 ( 2.2) | 10 ( 7.5) |  |
|  | 5=Caribbean (e.g., Jamaican, Cuban, Haitian, Trinidad and Tobago, etc.) | 20 ( 9.0) | 8 ( 9.0) | 12 ( 9.0) |  |
|  | 6=Middle Eastern/West Asian (e.g., Iranian, Afghan, Lebanese, etc.) | 3 ( 1.3) | 1 ( 1.1) | 2 ( 1.5) |  |
|  | 7=Southeast Asian (e.g., Vietnamese, Cambodian, Laotian, Thai, Filipino, etc.) | 7 ( 3.1) | 5 ( 5.6) | 2 ( 1.5) |  |
|  | 8=Latin American (e.g., Brazilian, Guatemalan, Colombian, etc.) | 5 ( 2.2) | 2 ( 2.2) | 3 ( 2.2) |  |
|  | 9=Indigenous/Aboriginal | 6 ( 2.7) | 3 ( 3.4) | 3 ( 2.2) |  |
|  | 10=European (e.g., German, Ukrainian, Portuguese, etc.) | 42 (18.8) | 16 (18.0) | 26 (19.4) |  |
|  | 11=United Kingdom (e.g., England, Scotland, Ireland etc.) | 2 ( 0.9) | 1 ( 1.1) | 1 ( 0.7) |  |
|  | 12=Mixed background | 51 (22.9) | 17 (19.1) | 34 (25.4) |  |
|  | 13=Other | 9 ( 4.0) | 2 ( 2.2) | 7 ( 5.2) |  |
|  | 999=No information | 14 ( 6.3) | 6 ( 6.7) | 8 ( 6.0) |  |
